# Synthesis of Ru alkylidene complexes

**DOI:** 10.3762/bjoc.7.14

**Published:** 2011-01-21

**Authors:** Renat Kadyrov, Anna Rosiak

**Affiliations:** 1 Evonik Degussa GmbH, Rodenbacher Chaussee 4, 63457 Hanau-Wolfgang, Germany; 2 present address: ASM Research Chemicals, Feodor-Lynen-Str. 31, 30625 Hannover, Germany

**Keywords:** alkylidene complexes, metathesis, rotation barrier, ruthenium

## Abstract

The present work describes the robust synthesis of Ru alkylidene complexes (PCy_3_)_2_Cl_2_Ru=CHR – precursors for metathesis catalysts. Moreover, the dynamic behavior of complexes where R = 2-naphthyl and 2-thienyl was studied. ^1^H NMR techniques were employed to establish the preferred conformations in solution for both complexes and the energy barrier for rotation around single (Ru=)CH–C(thienyl) bond was estimated (Δ*G*^≠^_303K_ = 12.6 kcal/mol).

## Introduction

The key to active ruthenium metathesis initiators is the accessibility of the ruthenium precursor containing the alkylidene moiety. The most commonly used precursors for the “second generation” catalysts bearing NHC ligands are the alkylidene ruthenium complexes coordinated with two phosphines [1]. For recent reviews see [2–4]. There are several routes for accessing five-coordinated ruthenium(II) alkylidene complexes such as diazo-transfer [5] and the reaction of vinyl or propargyl halides with hydrido(dihydrogen)-Ru-complexes generated from [Ru(COD)Cl_2_] and PCy_3_ under hydrogen pressure [6]. It should also be noted that the method for the generation of such highly reactive hydrido(dihydrogen)-Ru-complexes was first described by Werner and co-workers who employed two equivalents of *i*Pr_3_P in 2-butanol and hydrogen [7]. This last attractive one-pot procedure without the use of hydrogen was improved by the Ciba-group [8–9]. Werner and co-workers also published a one-pot synthesis of the complex (PCy_3_)_2_Cl_2_Ru=CHMe (**1a**) by direct reduction of RuCl_3_ with Mg/ClCH_2_CH_2_Cl in THF in the presence of excess PCy_3_ and hydrogen followed by subsequent reaction with acetylene [10].

We report herein on an improved protocol for the synthesis of the ethylidene complex (PCy_3_)_2_Cl_2_Ru=CHMe (**1a**) under mild conditions which is an efficient precursor for the preparation of wide variety of other alkylidene complexes.

## Results and Discussion

**Scheme 1:**
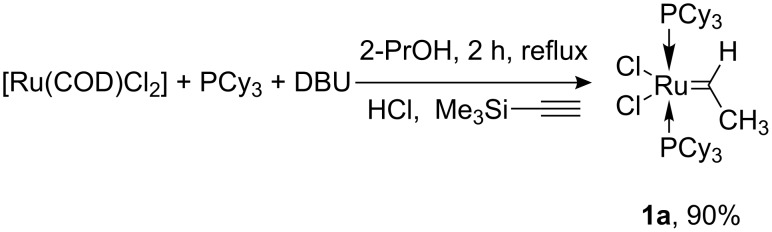
Synthesis of complex **1a**.

Van der Schaaf and co-workers published in 2000 a simple one-pot procedure for the synthesis of the ruthenium benzylidene complex (*i*Pr_3_P)_2_Cl_2_Ru=CHPh [8]. It was mentioned that also (PCy_3_)_2_Cl_2_Ru=CHPh could be similarly prepared. To our surprise, by following exactly the given protocol using DBU as base, a mixture of the desired benzylidene complex (PCy_3_)_2_Cl_2_Ru=CHPh together with the vinylidene complex (PCy_3_)_2_Cl_2_Ru=C=CHPh was obtained. Obviously, the last complex originated from reaction of an intermediate hydride species with phenyl acetylene along with formation of the benzylmethylidene complex (PCy_3_)_2_Cl_2_Ru=CHCH_2_Ph as described previously by Werner [7]. We have found that the use of trimethylsilylacetylene afforded the ethylidene complex **1a** as the sole product in very good isolated yield (see Scheme 1).

**Scheme 2:**
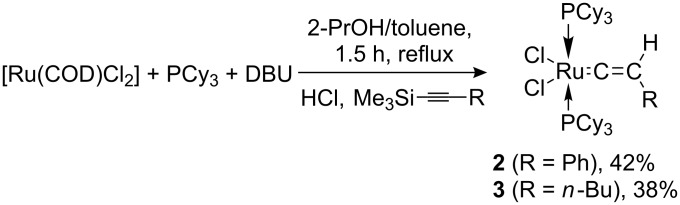
Synthesis of complexes **2** and **3**.

In sharp contrast, the use of 1-phenyl-2-trimethylsilylacetylene or 1-trimethylsilyl-1-hexyne gave the vinylidene complexes **2** and **3** in only moderate isolated yields (see Scheme 2).

**Scheme 3:**
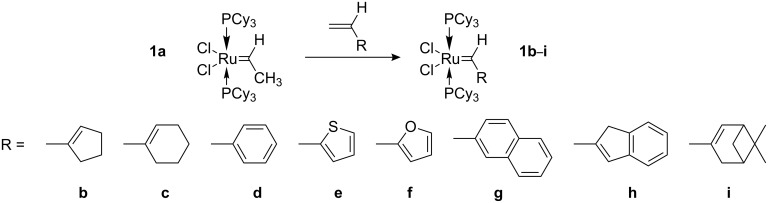
Synthesis of complexes **1b**–**i**.

Compound **1a** is remarkably stable below room temperature and readily exchanges the ethylidene moiety with other alkenes. Thus, compound **1a** is an ideal precursor for a variety of other ruthenium alkylidene complexes. Compounds **1b**–**i** (Scheme 3) were readily isolated and characterized spectroscopically. It is noteworthy, that with the exception of **1e** and **1g**, all isolated complexes decompose slowly in chlorinated organic solvents. Therefore, cross metatheses in toluene in general led to alkylidene complexes with higher isolated yields.

**Figure 1:**
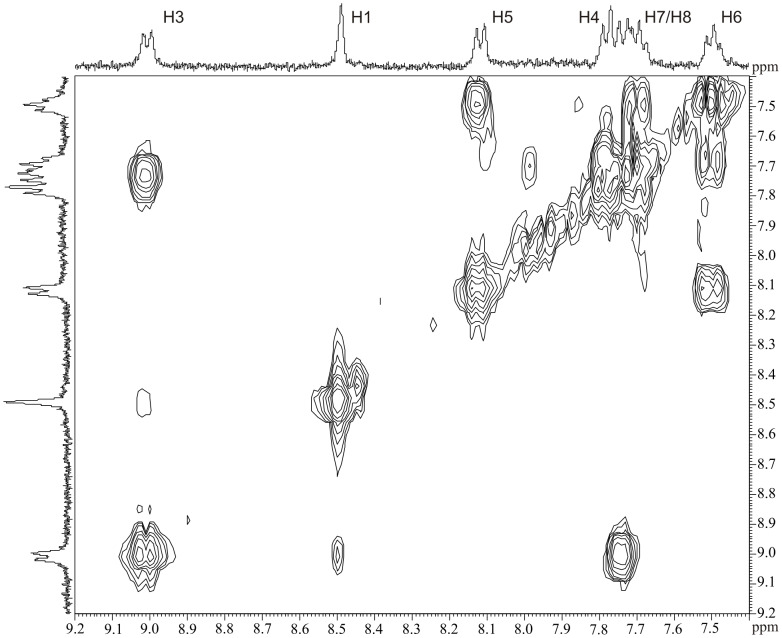
Naphthyl-group region of ^1^H,^1^H-COSY NMR for **1g** in CD_2_Cl_2_ at −80 °C.

The NMR spectra of compounds **1b,c,e**–**i** displayed more or less broad signals at ambient temperature. In particular, lowering the temperature of solutions of **1e** and **1g** in CD_2_Cl_2_ caused further broadening of the NMR resonances which only become properly resolved for the aromatic and methylidene signals at −80 °C. The slow exchange resonances of compound **1g** are better resolved due to the lower concentration of the minor isomer. A ^1^H,^1^H-COSY experiment at −80 °C enabled the identification of the aromatic resonances in the low temperature spectrum (Figure 1). The singlet at 8.49 ppm is assigned to H1 and the doublet at 9.01 ppm to H3 on the basis of the observed weak coupling ^4^*J*(H1H3). The strong coupling of ^3^*J*(H3H4) = 8.2 Hz with doublet at 9.01 ppm allows the assignment of H4 (7.78 ppm). Other coupling patterns are consistent with the resonances of the residual protons H5 (8.12, d, *J* = 8.2 Hz), H6 (7.50, t, *J* = 7.0 Hz) and H7/H8 (7.67-7.75, m).

**Figure 2:**
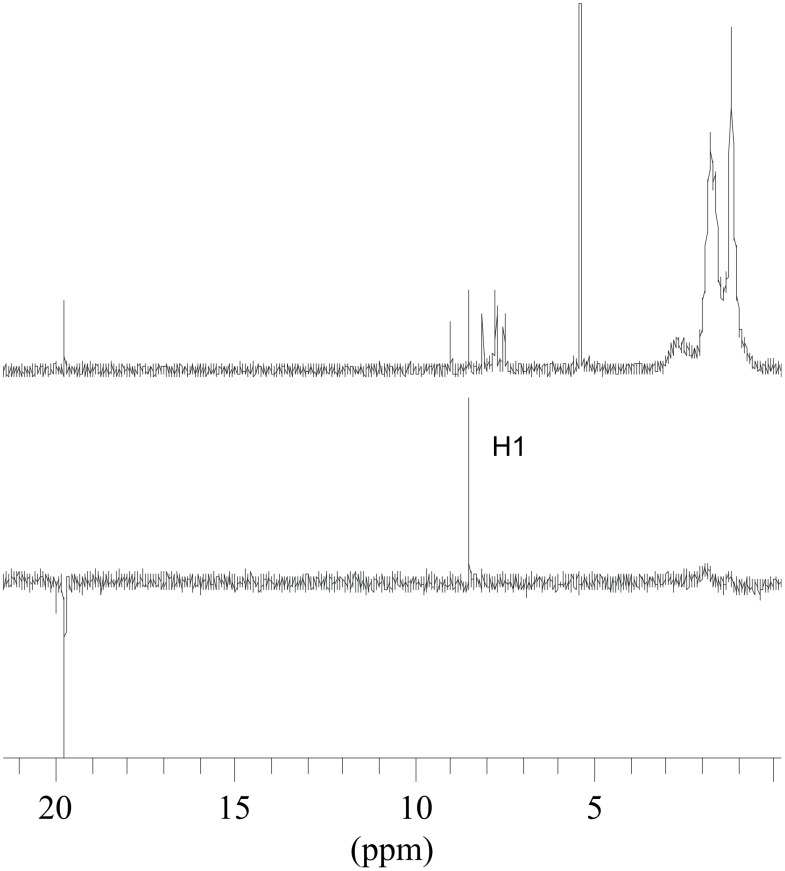
^1^H NMR (top) and NOE difference spectrum (bottom) of **1g** in CD_2_Cl_2_ at −80 °C, saturating the methylidene H signal at δ = 19.75 ppm.

**Scheme 4:**
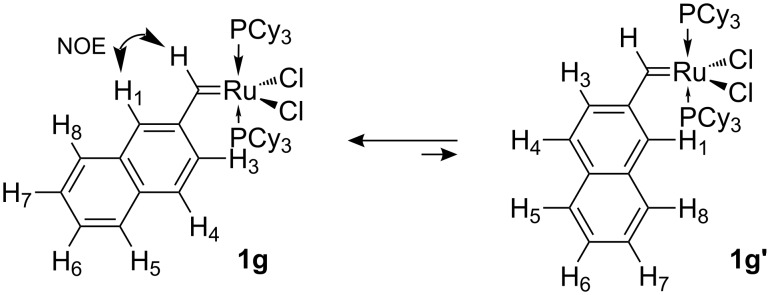
Conformational isomerism in complex **1g**.

Strong NOE enhancement of H1 upon saturation of the carbene proton at 19.75 ppm (see Figure 2) is consistent with preferred conformer **1g** in which the naphthyl moiety is directed away from the phosphine ligand (see Scheme 4).

**Figure 3:**
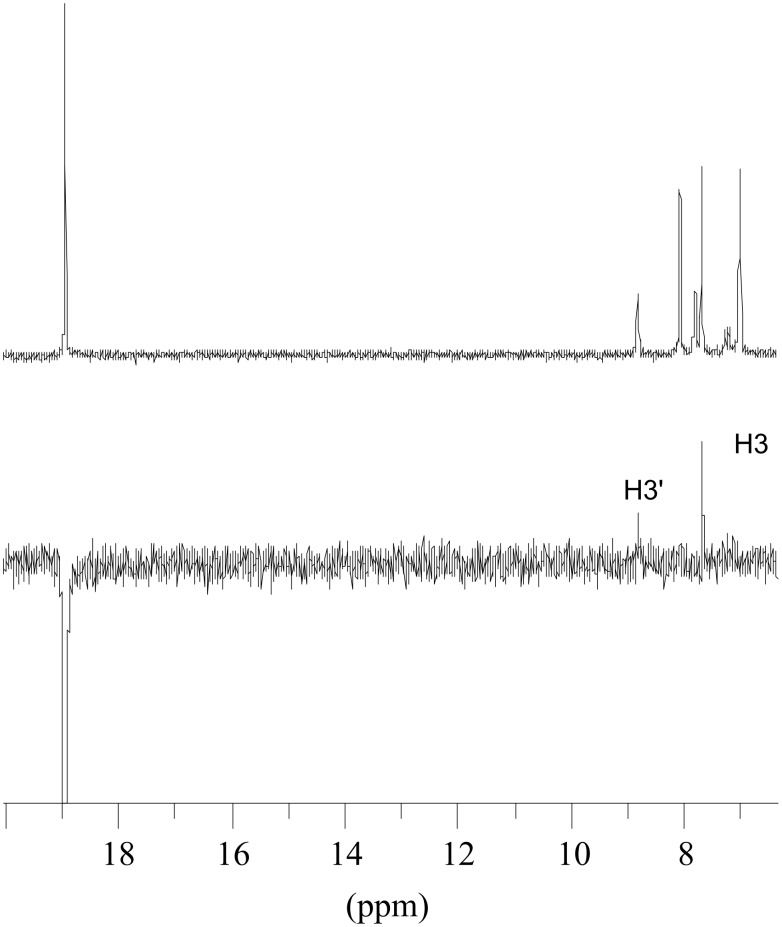
Olefin and alkylidene-proton region of the ^1^H NMR (top) and NOE difference spectrum (bottom) of **1e** in CD_2_Cl_2_ at −40 °C, saturating the methylidene H signal at δ = 18.9 ppm.

**Scheme 5:**
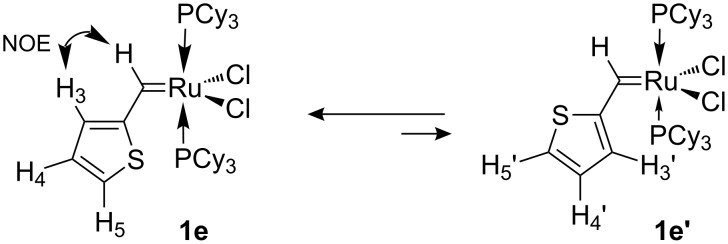
Conformational isomerism in complex **1e**.

**Figure 4:**
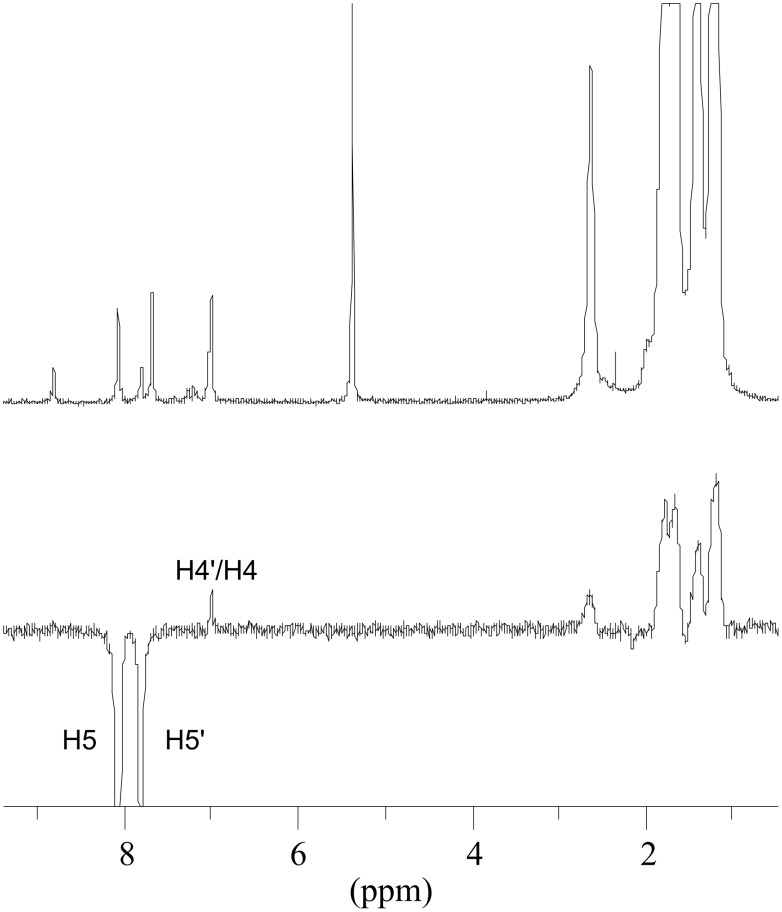
Olefin and alkyl group region of the ^1^H NMR (top) and NOE difference spectrum (bottom) of **1e** in CD_2_Cl_2_ at −40 °C, saturating the thienyl-H5 signal at δ = 8.07 ppm.

At low temperature both isomers of **1e** are visible in the NMR spectrum due to comparable concentrations (obtained enthalpy difference Δ*H* = 1.3 kcal/mol, see Supporting Information File 1). A number of NOE experiments at −40 °C allowed the assignment of the resonances of both isomers **1e** and **1e’**. Saturation of the carbene proton at 18.9 ppm led to strong NOE enhancement of the singlet at 7.68 ppm (Figure 3) and allowed the assignment of this signal to the H3 proton of the thienyl moiety and was consistent with the *s-trans* isomer **1e** being the preferred conformer (see Scheme 5). The EXSY effect made it possible to assign the signal at 8.80 ppm to H3’ of the minor *s-cis* conformer. Enhancement of the signal at 6.99 ppm (Figure 4) by saturation of the signal at 8.07 ppm and EXSY inversion of the resonance at 7.79 ppm allowed the assignment of the signals for H5 (8.07 ppm), H4 (6.99 ppm), H5’ (7.79 ppm) and H4’ (7.03 ppm).

The thiophene region of the ^1^H NMR spectrum of **1e** was simulated and iteratively fitted to the experimental spectra in order to evaluate the rate constants at various temperatures (Supporting Information File 1). Linear regression analysis of these data gave activation enthalpy Δ*H*^≠^= 13.7 kcal/mol. From the rate constant at 303 K the value of free energy of activation (Δ*G*^≠^_303K_ = 12.6 kcal/mol) was also calculated. This is substantially higher than several calculated (*E*_a_ = 4.4 kcal/mol) [11–12] and experimentally estimated (*E*_a_ = 5.7 kcal/mol) [13] internal rotation barriers of styrene, 2-vinylthiophene (*E*_a_ = 4.8 kcal/mol) [14] and is comparable with rotation barrier of the aryl ring in chromium carbene complexes (Δ*G*^≠^_298K_ = 13.0–16.2 kcal/mol) [15].

## Experimental

Routine, 2D-correlation spectra (^1^H,^1^H-COSY) and SELNOE experiments were recorded on a Bruker Avance-400 (BPFO-Plus with Z gradient) spectrometers. ^1^H NMR chemical shifts are reported in ppm relative to TMS at 0 ppm. IR spectra were recorded on a Tensor 27 FT-IR Spectrometer (Bruker) with MKII Golden Gate Single Reflection Diamond ATR System. For ESI-MS spectra, a Q-TOF Premier (Waters) was used. All solvents used were anhydrous grade purchased from Aldrich. Commercially available compounds were used without further purification. 2-Vinylthiophene [16], 2-vinylfuran [17], 1-vinylcyclohexene [18], 1-vinylcyclopentene [18] and nopadiene [19] were prepared according to known procedures.

**2-Vinylindene was prepared by a slightly modified literature procedure** [18]**:** A solution of 2-indanone (5 g, 38 mmol) in dry THF (10 mL) was added over 10 min to a cooled (ice bath) and stirred solution of vinylmagnesium chloride (1.6 M in THF, 36 mL, 57 mmol). The mixture was stirred at 60 °C for a further 30 min and then cooled, quenched with saturated NH_4_Cl solution, and finally extracted thoroughly with ether. The combined organic extracts were washed with brine, dried, and concentrated at reduced pressure. The residue was dissolved in pyridine (30 mL). POCl_3_ (4.5 mL, 45 mmol) was slowly added to this solution at 4 °C under an argon atmosphere. The resulting mixture was stirred for further 10 h in an ice bath and then slowly allowed to warm to ambient temperature overnight. The resulting dark brown mixture was poured into ice water and the product extracted with ether. The extracts were washed successively with 2N HCl and then brine. After drying and filtration through a short pad of silica gel, the crude product was purified by distillation to yield 2-vinylindene (2.59 g, 18.2 mmol, 48%) as a colorless liquid, bp 93–94 °C/200 mbar. ^1^H NMR (CDCl_3_): δ = 7.38 (d, *J* = 7.1 Hz, 1H), 7.30 (d, *J* = 7.4 Hz, 1H), 7.21 (dd, *J* = 8.0 Hz, *J* = 7.5 Hz, 1H), 7.21 (dt, *J* = 1.1 Hz, *J* = 7.4 Hz, 1H), 6.74 (dd, *J* = 17.5 Hz, *J* = 10.6 Hz, 1H), 6.71 (s, 1H), 5.41 (d, *J* = 17.4 Hz, 1H), 7.30 (d, *J* = 10.6 Hz, 1H), 3.52 (s, 2H) ppm.

**Dichlorobis(tricyclohexylphosphine)(ethylidene)ruthenium(II)** (**1a**)**:** 1,8-Diazabicyclo[5.4.0]undec-7-ene (3.3 mL, 22 mmol) and tricyclohexylphosphine (6.17 g, 22 mmol) were added under an argon atmosphere to a suspension of dichloro(1,5-cyclooctadiene)ruthenium(II) (2.8 g, 10 mmol) in isopropanol (100 mL). The resulting mixture was heated at reflux for 2 h. THF (150 mL) was added to the resulting brick-red suspension which was allowed to cool to 15 °C prior to the addition of 2M HCl in ether (12 mL). After stirring for 5 min, trimethylsilylacetylene (4.2 mL, 30 mmol) was added and the resulting purple colored mixture stirred in an ice bath for 3 h. THF was then evaporated at 4 °C in order to complete the precipitation. The solid product was filtered by suction, washed thoroughly with chilled methanol and vacuum dried at 0–5 °C to give 6.85 g (90%) of purple crystals. ^31^P NMR (CDCl_3_): δ *=* 35.8 ppm; ^1^H NMR (CDCl_3_): δ = 19.30 (q, *J* = 5.6 Hz, 1H), 2.60 (d, *J* = 5.5 Hz, 3H), 2.60–2.52 (m, 6H), 1.88–1.22 (m, 60H) ppm.

**General procedure A for the synthesis of alkylidene complexes:** (PCy_3_)Cl_2_Ru=CHMe (**1a**) (1 mmol) was added to a stirred and cooled (ice bath) solution containing a four-fold excess of the respective olefin in degassed CH_2_Cl_2_ (25 mL). Argon was bubbled through the resulting dark violet solution for 2 h at 4 °C and then for a further 30 min at room temperature. The reaction mixture was again chilled in ice bath. Degassed methanol (20 mL) was added and the CH_2_Cl_2_ removed in vacuo at 0–5 °C. To complete the precipitation another portion of degassed chilled methanol (10 mL) was added and the precipitated product was filtered by suction. The resulting solid was washed thoroughly with chilled methanol, sucked as dry as possible, washed with hexane and dried under vacuum.

**General procedure B for the synthesis of alkylidene complexes:** (PCy_3_)Cl_2_Ru=CHMe (**1a**) (1 mmol) was added to a stirred and cooled (ice bath) solution containing a four-fold excess of the respective olefin in degassed toluene (25 mL). Argon was bubbled through the resulting dark violet solution for 2 h at 4 °C and then for a further 30 min at room temperature. Toluene was removed in vacuum at 20 °C and the residue triturated with chilled methanol (20 mL). The precipitated product was filtered by suction, washed thoroughly with chilled methanol and dried under vacuum.

**Dichlorobis(tricyclohexylphosphine)(cyclopenten-1-ylmethylidene)ruthenium(II)** (**1b**): The product (violet solid) was prepared according to general procedure B in 80% yield. ^31^P NMR (CDCl_3_): δ *=* 37.26 ppm; ^1^H NMR (CDCl_3_): δ = 19.30 (s, 1H), 6.97 (s, 1H), 3.14 (m, 2H), 2.60 (m, 6H), 1.95–1.11 (m, 64H) ppm. ^13^C NMR: δ = 285.83, 164.61, 139.83, 36.97, 34.80; 31.95 (t, *J* = 9.1), 29.63, 27.91 (t, *J* = 5.0), 26.64, 25.15.

**Dichlorobis(tricyclohexylphosphine)(cyclohexen-1-ylmethylidene)ruthenium(II)** (**1c**): The product as a toluene adduct (intensive violet solid) was prepared according to general procedure B in 46% yield. ^31^P NMR (C_6_D_6_): δ *=* 36.53 ppm; ^1^H NMR (C_6_D_6_): δ =19.08 (s, 1H), 7.21 (s, 1H), 2.87 (m, 2H), 2.60 (m, 6H), 1.95–1.11 (m, 66H) ppm. ^13^C NMR: δ = 296.40 (d, *J* = 113.4), 157.46, 140.27, 32.08 (t, *J* = 9.1); 30.28, 29.99, 29.70; 27.93 (t, *J* = 5.0), 27.93, 26.67, 22.97, 21.45. Toluene 137.82, 129.05, 128.24, 125.31, 21.41.

**Dichlorobis(tricyclohexylphosphine)(benzylidene)ruthenium(II)** (**1d**): The product (violet solid) was prepared according to general procedure A in 81% yield. The NMR spectra were in agreement with the spectra reported in the literature [5].

**Dichlorobis(tricyclohexylphosphine)(thien-2-ylmethylidene)ruthenium(II)** (**1e**): The product (dark violet solid) was prepared according to general procedure A in 71% yield. ^31^P NMR (CDCl_3_): δ *=* 35.96 ppm; ^1^H NMR (CD_2_Cl_2_): δ = 19.05 (s, 1H), 8.09 (s, br., 1H), 7.84 (d, *J* = 4.1 Hz, 1H), 6.90 (t, *J* = 4.3 Hz, 1H), 2.55 (m, 6H), 1.75–1.60 (m, 30H), 1.39–1.35 (m, 12H), 1.20–1.12 (m, 18H) ppm. ^13^C NMR (CDCl_3_): δ = 269.11, 163.84 (br.), 133.09 (br.), 129.22, 32.26 (t, *J* 0 9.1), 29.68, 27.85 (t, *J* = 5.0), 26.55. IR (ATR): λ^−1^ = 2919 (vs), 2848 (s), 2169 (w), 2051 (w), 1936 (w), 1901 (w), 1443 (m), 1403 (m), 1353 (m), 1263 (m), 1005 (m), 734 (vs) cm^−1^. MS(ESI): m/z (%) = 828 (21) [M^+^], 793 (9), 281 (100).

**Dichlorobis(tricyclohexylphosphine)(fur-2-ylmethylidene)ruthenium(II)** (**1f**): The product (dark violet solid) was prepared according to general procedure A in 56% yield. ^31^P NMR (CDCl_3_): δ *=* 37.04 ppm; ^1^H NMR (CDCl_3_): δ = 18.79 (s, 1H), 8.12 (s, br., 1H), 7.74 (s, br., 1H), 6.43 (dd, *J* = 3.6 Hz, *J* = 1.7, 1H), 2.64 (m, 6H), 1.81–1.67 (m, 30H), 1.48–1.41 (m, 12H), 1.27–1.14 (m, 18H) ppm. ^13^C NMR: δ = 259.90 (d, *J* = 105.1), 172.34 (br.), 141.71 (br.), 121.54 (br.), 115,44, 32,11 (t, *J* = 9.0), 29.62, 27.85 (t, *J* = 5.1), 26.56.

**Dichlorobis(tricyclohexylphosphine)(naphth-2-ylmethylidene)ruthenium(II)** (**1g**): The product (dark violet solid) was prepared according to general procedure A in 56% yield. ^31^P NMR (CDCl_3_): δ *=* 37.43 ppm; ^1^H NMR (CDCl_3_): δ = 20.12 (s, 1H), 8.82 (s, br., 1H), 8.77 (d, *J* = 8.5 Hz, 1H), 8.06 (d, *J* = 8.1 Hz, 1H), 7.74 (d, *J* = 8.1 Hz, 1H), 7.71 (d, *J* = 8.4 Hz, 1H), 7.67–7.63 (m, 1H), 7.46–7.42 (m, 1H), 2.63 (m, 6H), 1.90–1.60 (m, 30H), 1.46–1.37 (m, 12H), 1.30–1.10 (m, 18H) ppm. ^13^C NMR: δ = 292.71, 150.48, 133.98, 133.11, 130.56, 129.77, 129.04, 128.35, 128.05, 127.23, 126.86, 32.19 (t, *J* = 9.1), 29.70, 27.85 (t, *J* = 5.1), 26.54. IR (ATR): λ^−1^ = 2922 (vs), 2848 (s), 2358 (w), 2003 (w), 1443 (m), 1265 (m), 1004 (m), 733 (vs) cm^−1^. MS (ESI): m/z (%) = 872 (2) [M^+^], 333 (100).

**Dichlorobis(tricyclohexylphosphine)(inden-2-ylmethylidene)ruthenium(II)** (**1h**): The product (brick-red solid) was prepared according to general procedure A in 37% yield. ^31^P NMR (CDCl_3_): δ *=* 36.93 ppm; ^1^H NMR (CDCl_3_): δ = 19.64 (s, 1H), 7.94 (s, br., 1H), 7.73 (d, *J* = 7.7 Hz, 1H), 7.45 (m, 3H), 4.23 (s, 2H), 2.63 (m, 6H), 1.81 (d, *J* = 12.0 Hz, 18H), 1.70 (dd, *J* = 23.7, *J* = 11.9, 12H), 1.47 (dd, *J* = 23.7 Hz, *J* = 11.9 Hz, 12H), 1.28–1.17 (m, 18H) ppm.

**Dichlorobis(tricyclohexylphosphine)(norpinanylmethylidene)ruthenium(II)** (**1i**): The product (violet solid) was prepared according to general procedure B in 43 % yield. ^31^P NMR (CDCl_3_): δ *=* 36.54 ppm; ^1^H NMR (CDCl_3_): δ = 19.12 (s, 1H), 7.65 (s, 1H), 3.02 (m, 1H), 2.48 (m, 6H), 2.30 (m, 1H), 2.09–1.12 (m, 67H), 0.70 (s, 3H) ppm. ^13^C NMR: δ = 291.46, 163.88, 134.87, 49.14, 39.66, 38.84, 34.77, 31.98 (t, *J* = 9.0), 31.84, 29.72 (d, *J* = 11.0), 27.93 (t, *J* = 4.7), 26.60, 26.45, 20.77 ppm.

**Dichlorobis(tricyclohexylphosphine)(2-phenylvinylylidene)ruthenium(II)** (**2**): 1,8-Diazabicyclo[5.4.0]undec-7-ene (0.75 mL, 5.2 mmol) and a 20% solution of tricyclohexylphosphine in toluene (7.7 mL, 5.9 mmol) were added under an argon atmosphere to a suspension of dichloro(1,5-cyclooctadiene)ruthenium(II) (660 mg, 2.35 mmol) in isopropanol (20 mL). The mixture was heated at reflux under an argon atmosphere for 1 h. Toluene (24 mL) was added to the resulting brick-red suspension and the mixture heated for further 30 min at reflux and then allowed to cool to 5–10 °C. 1-Phenyl-2-trimethylsilylacetylene (1.4 ml, 7 mmol) was added followed 10 min later by HCl in ether (2M, 2.4 mL, 4.8 mmol). The resulting purple colored mixture was stirred at ambient temperature for 2h and then concentrated. The residue was treated with 40 mL of chilled methanol and the precipitated product was filtered by suction. The solid was washed thoroughly with chilled methanol and dried under vacuum at 0–5 °C to yield 826 mg (42%) of a violet solid. ^31^P NMR (CDCl_3_): δ *=* 22.54 ppm; ^1^H NMR (CDCl_3_): δ = 7.13 (t, *J* = 7.6 Hz, 2H), 6.89 (d, *J* = 7.5 Hz, 2H), 6.84 (t, *J* = 7.2 Hz, 1H), 4.35 (t, *J* = 3.3 Hz, 1H), 2.62 (m, 6H), 2.06 (d, *J* = 12.3 Hz, 12H), 1.66–1.73 (m, 18H), 1.59 (dd, *J* = 23.6 Hz, *J* = 11.9 Hz, 12H), 1.16–1.26 (m, 18H) ppm. These data are in agreement with the literature [20].

**Dichlorobis(tricyclohexylphosphine)(2-butylvinylidene)ruthenium(II)** (**3**): 1,8-Diazabicyclo[5.4.0]undec-7-ene (0.75 mL, 52 mmol) and 20% solution of tricyclohexylphosphine in toluene (7.7 mL, 5.9 mmol) were added under an argon atmosphere to a suspension of dichloro(1,5-cyclooctadiene)ruthenium(II) (660 mg, 2.35 mmol) in isopropanol (20 mL). The mixture was then heated at reflux under an argon atmosphere for 1 h. Toluene (24 mL) was added to the resulting brick-red suspension and the mixture heated for further 30 min at reflux and then allowed to cool to 5–10 °C. 1-Trimethylsilyl-1-hexyne (1.4 mL, 7 mmol) was added followed 10 min later by HCl in ether (2M, 2.4 mL, 4.8 mmol) and the resulting purple colored mixture stirred at ambient temperature for 2 h and then concentrated. The residue was treated with 40 mL of chilled methanol and the precipitated product was filtered by suction. The solid was washed thoroughly with chilled methanol and dried under vacuum to give 720 mg (38%) of a red-brown solid. ^31^P NMR (CDCl_3_): δ *=* 25.34 ppm; ^1^H NMR (CDCl_3_): δ = 3.41 (tt, *J* = 7.3 Hz, *J* = 1.7 Hz, 1H), 2.59 (m, 6H), 2.36 (dd, *J* = 14.0 Hz, *J* = 7.1 Hz, 2H), 2.06 (d, *J* = 12.1 Hz, 12H), 1.72–1.81 (m, 20H), 1.59 (dd, *J* = 22.7 Hz, *J* = 11.5 Hz, 12H), 1.16–1.26 (m, 22H), 0.87 (t, *J* = 6.8 Hz, 3H) ppm.

## Supporting Information

Features variable-temperature and simulated ^1^H NMR spectra of various compounds, Arrhenius plot of the equilibrium constants for **1e** and Eyring plot of the rate constants for **1e** interconversion.

File 1 Detailed experimental data.
