# Supplementary material for: Synthesis of Ru alkylidene complexes
Source: Beilstein J Org Chem. 2011 Jan 21;7:104–10. doi: 10.3762/bjoc.7.14 (PMC3028570; doi:10.3762/bjoc.7.14)

Supporting Information

for

Synthesis of Ru alkylidene complexes

Renat Kadyrov*[1] and Anna Rosiak[1,2]

Address: [1]Evonik Degussa GmbH, Rodenbacher Chaussee 4, 63457 Hanau-Wolfgang, Germany and [2]present address: ASM Research Chemicals, Feodor-Lynen-Str. 31, 30625 Hannover, Germany.

Email: renat.kadyrov@evonik.com

*Corresponding author

Detailed experimental data

Variable-temperature spectra of the solution of **1e** in CD_2Cl_2.

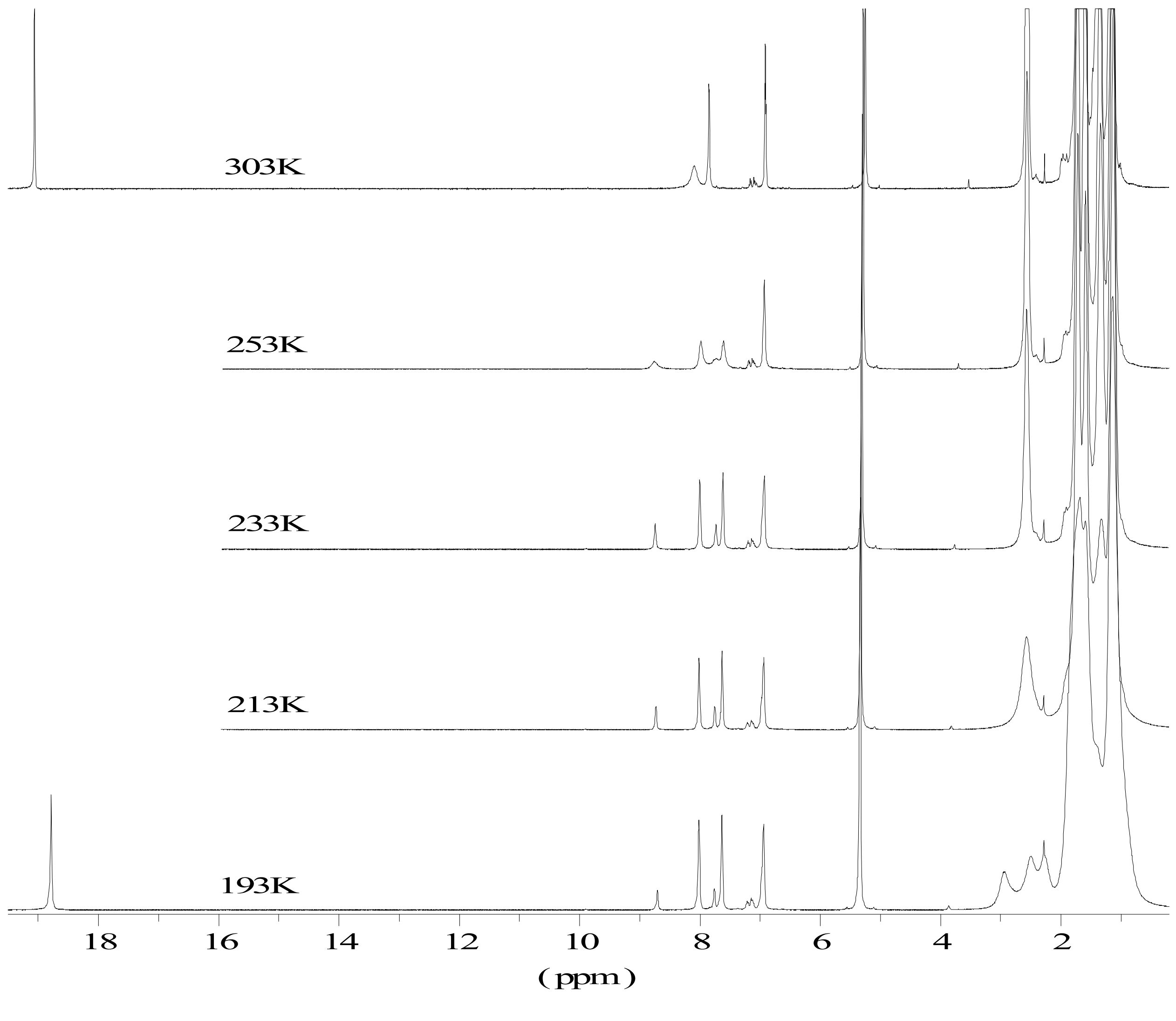

Experimetal (top) and calculated (bottom) spectrum parts (7.4-9.0 ppm) for exchanging signals H3', H5, H5' and H3 of the thienyl fragment in **1e**.

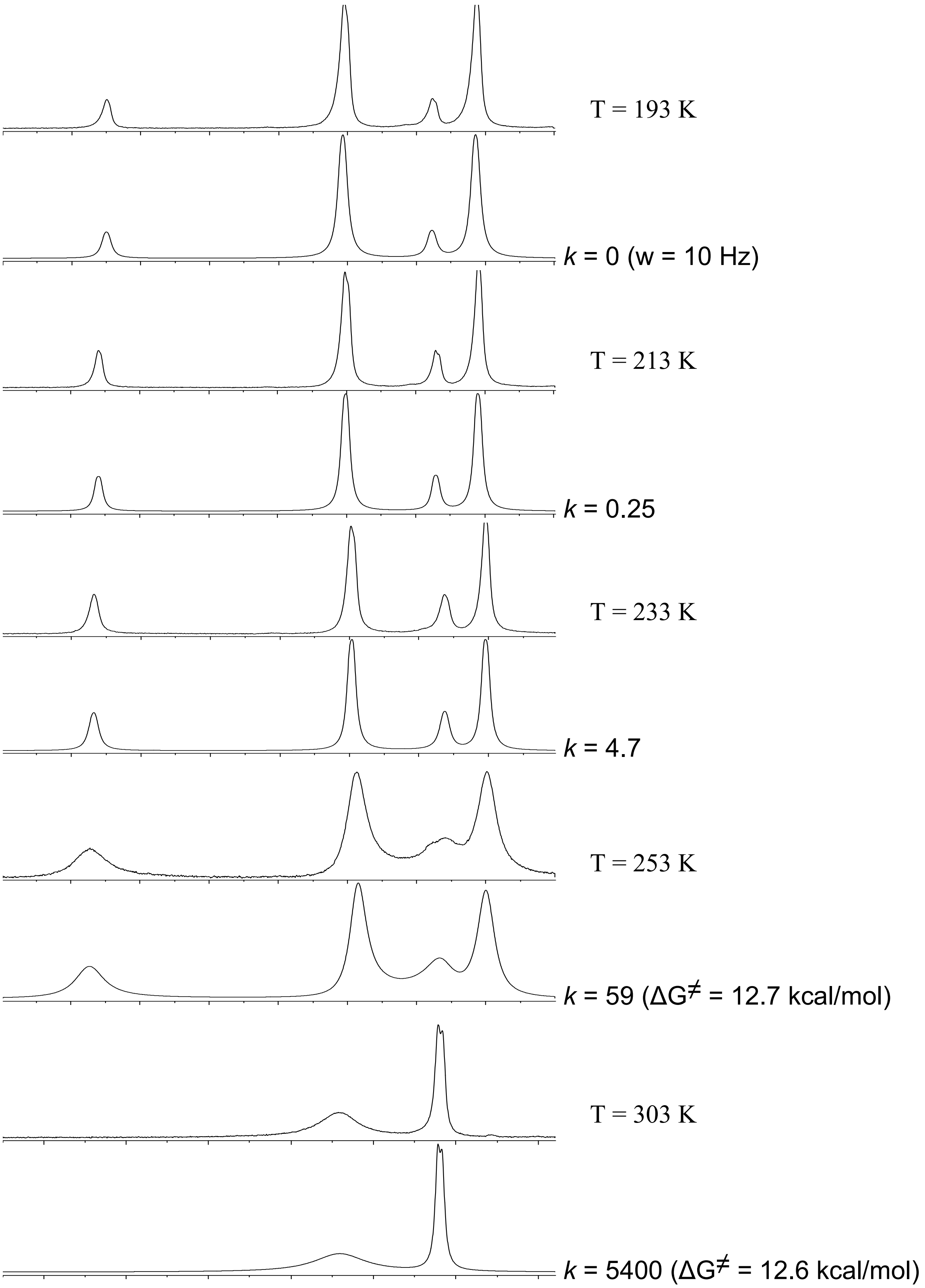

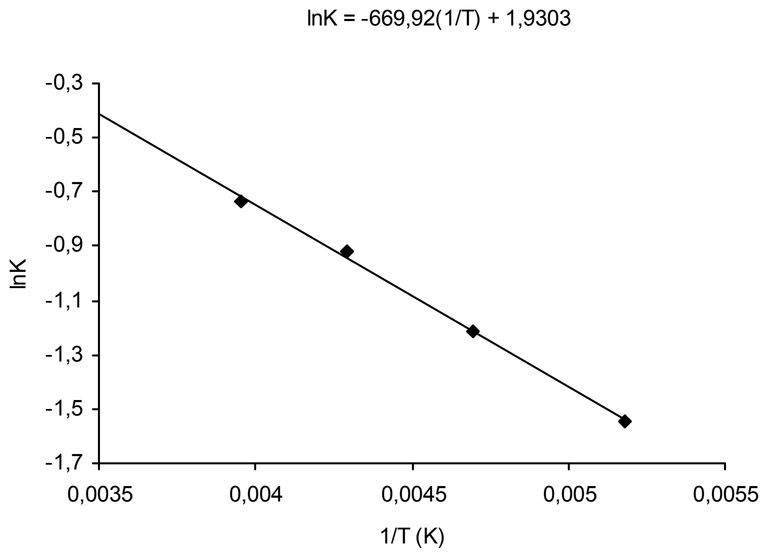

ΔH = 1.3 kcal/mol

Arrhenius plot for **1e** interconversion based on line-shape analysis.

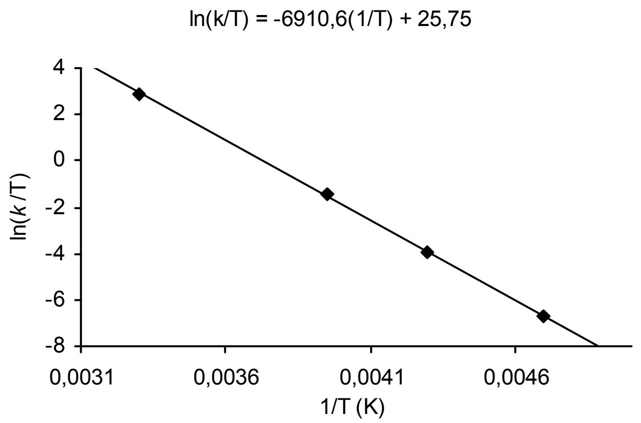

ΔH≠ = 13.7 kcal/mol

Eyring plot of the rate constants obtained from line shape analysis for **1e** interconversion.

Variable-temperature spectra of the solution of **1g** in CD_2Cl_2.

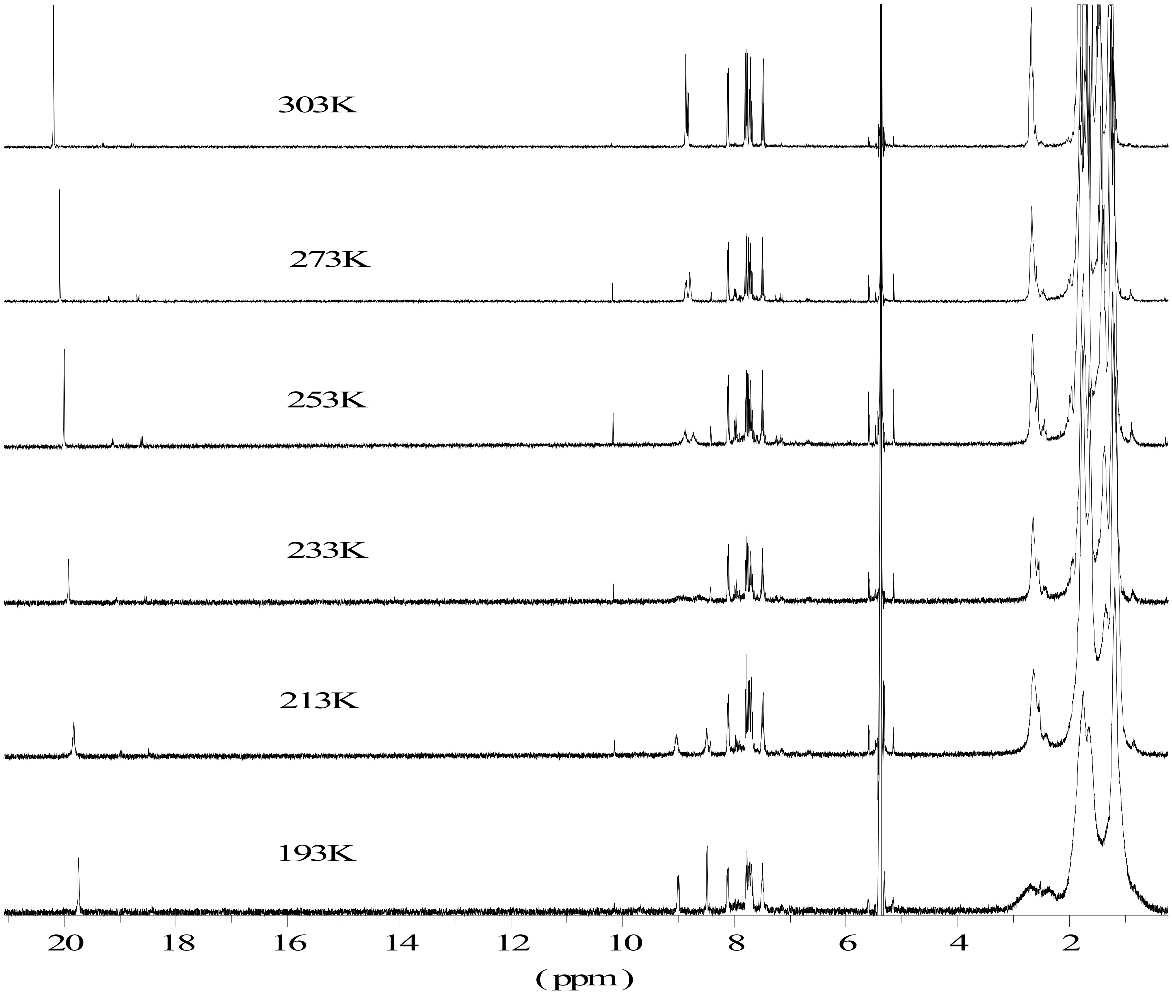

Dichlorobis(tricyclohexylphosphine)(ethylidene)ruthenium(II) (**1a**):

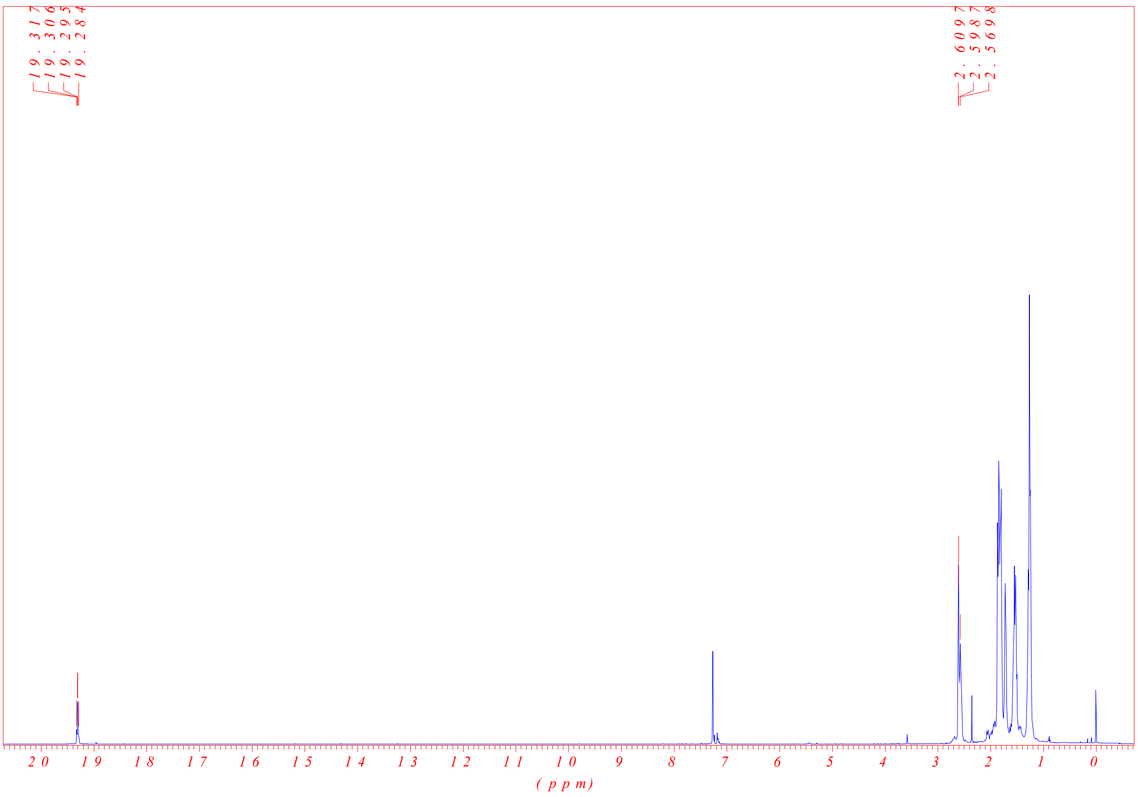

Dichlorobis(tricyclohexylphosphine)(cyclopenten-1-ylmethylidene)ruthenium(II) (**1b**):

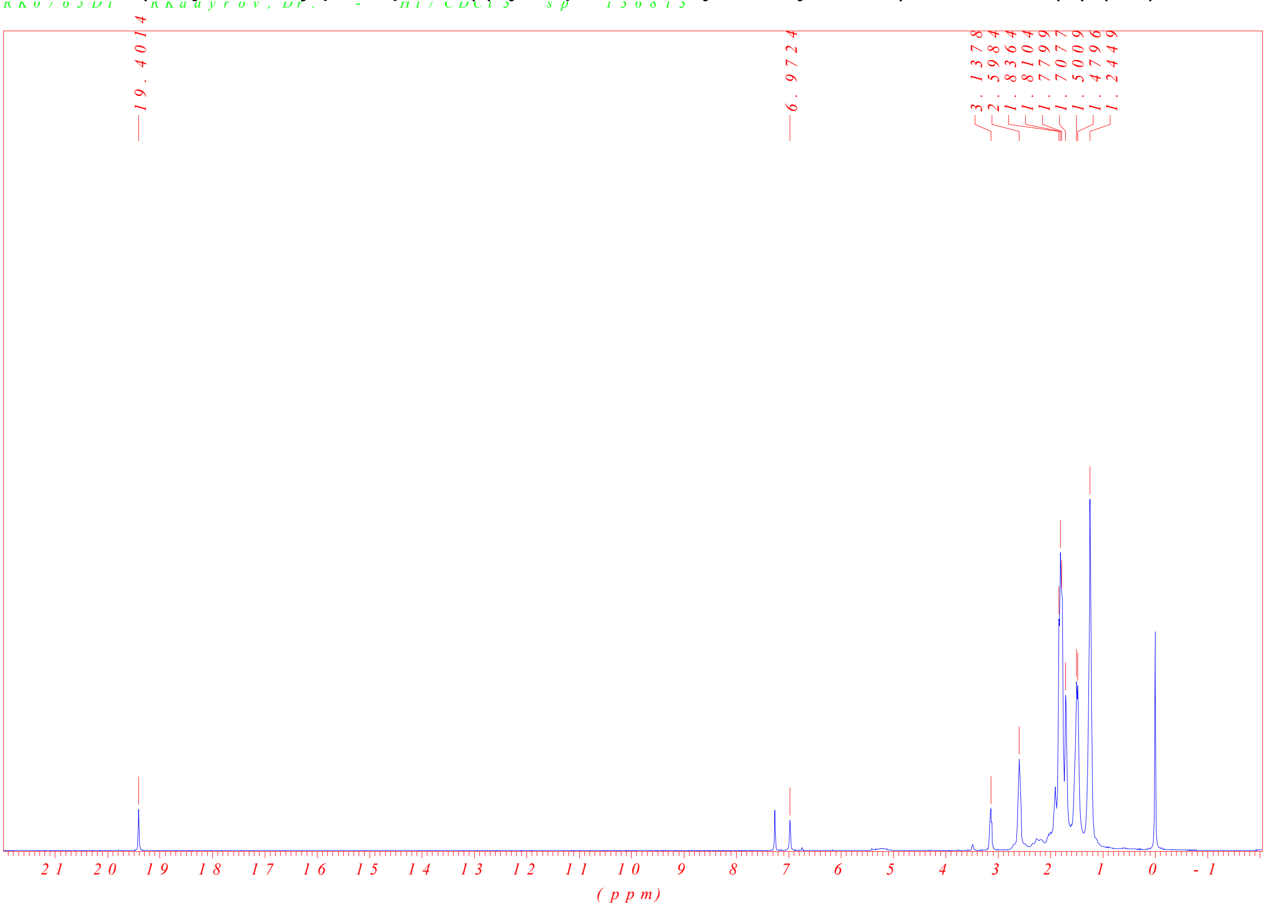

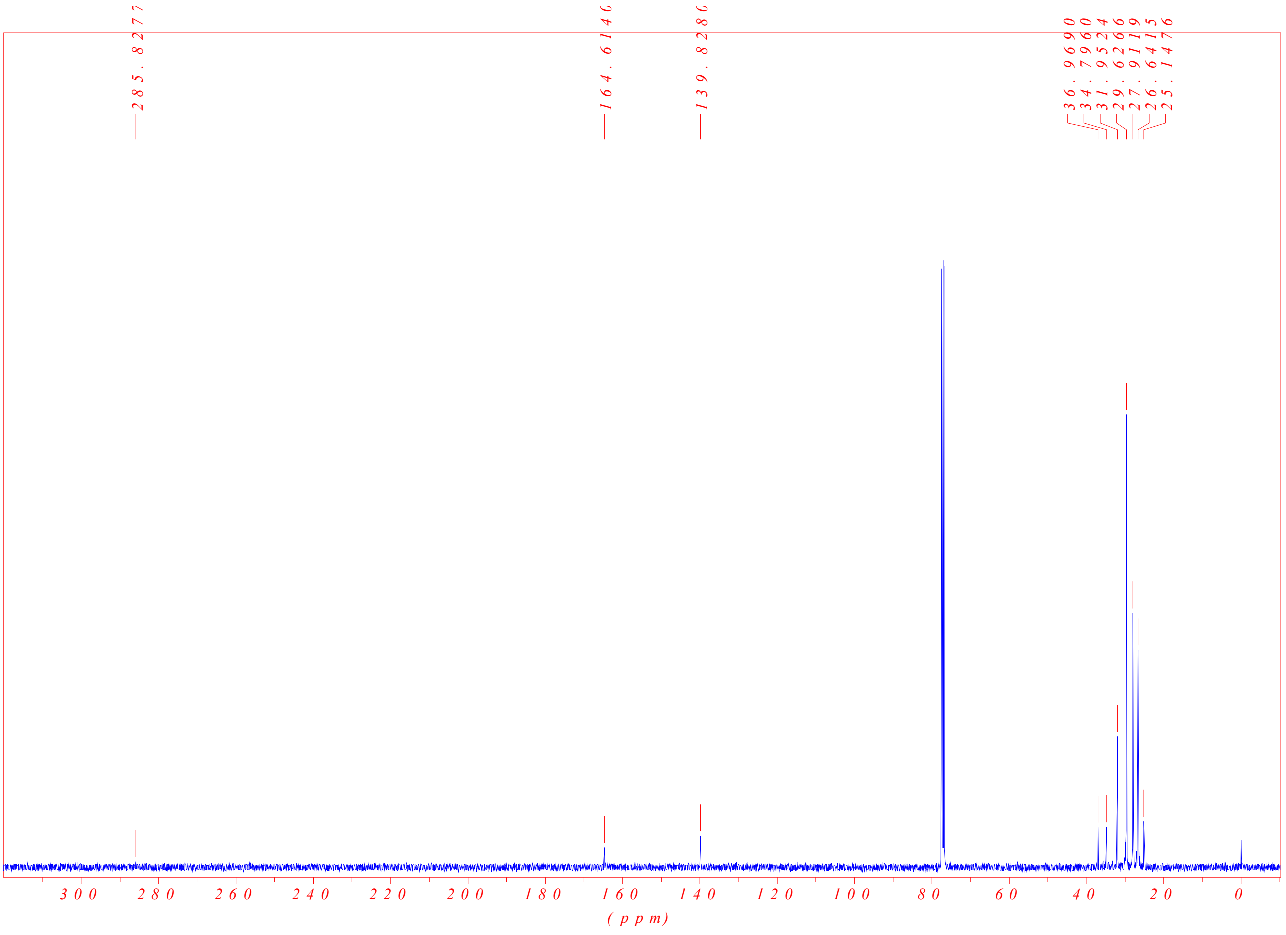

Dichlorobis(tricyclohexylphosphine)(cyclohexen-1-ylmethylidene)ruthenium(II)-toluene adduct (**1c·toluene**):

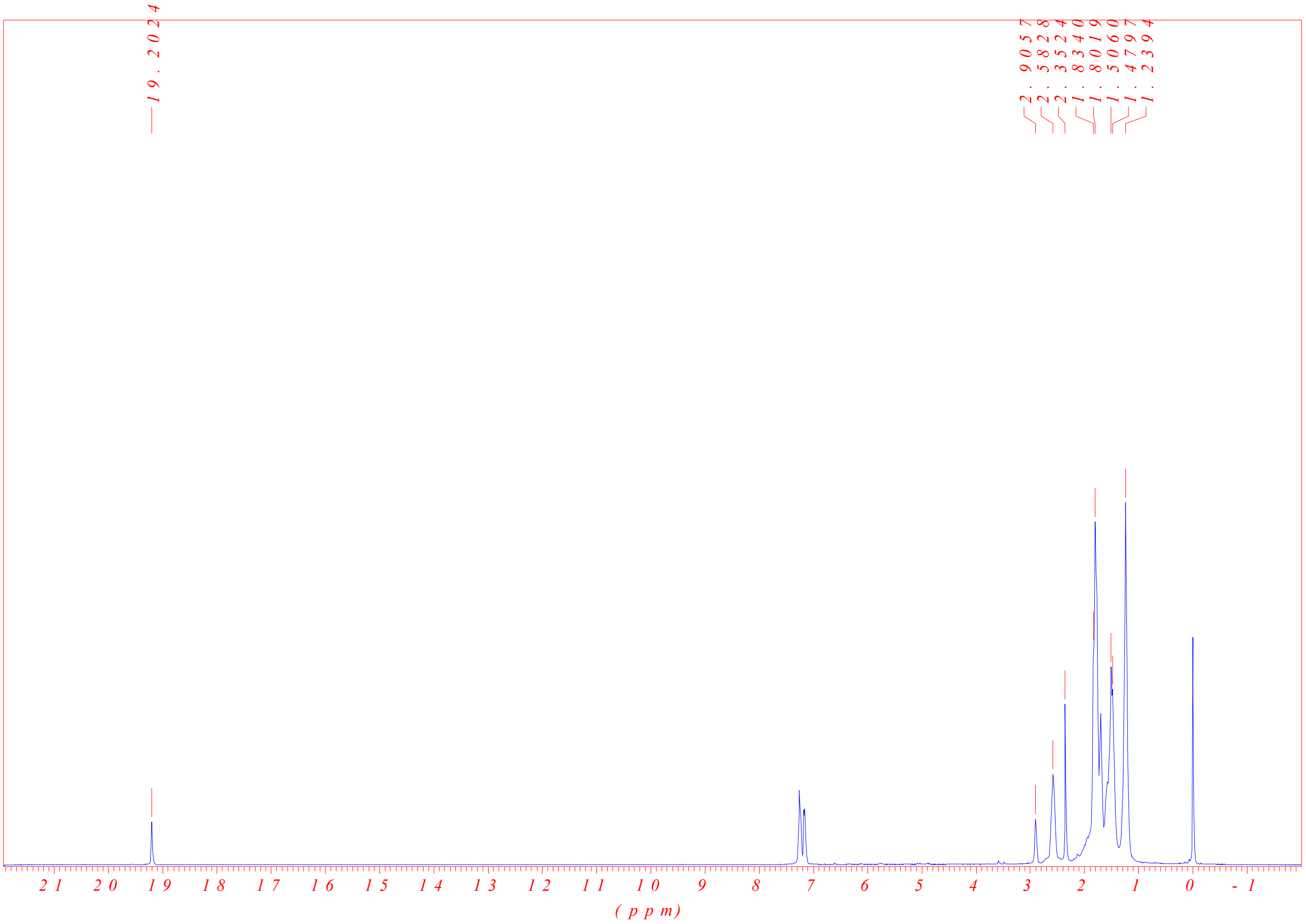

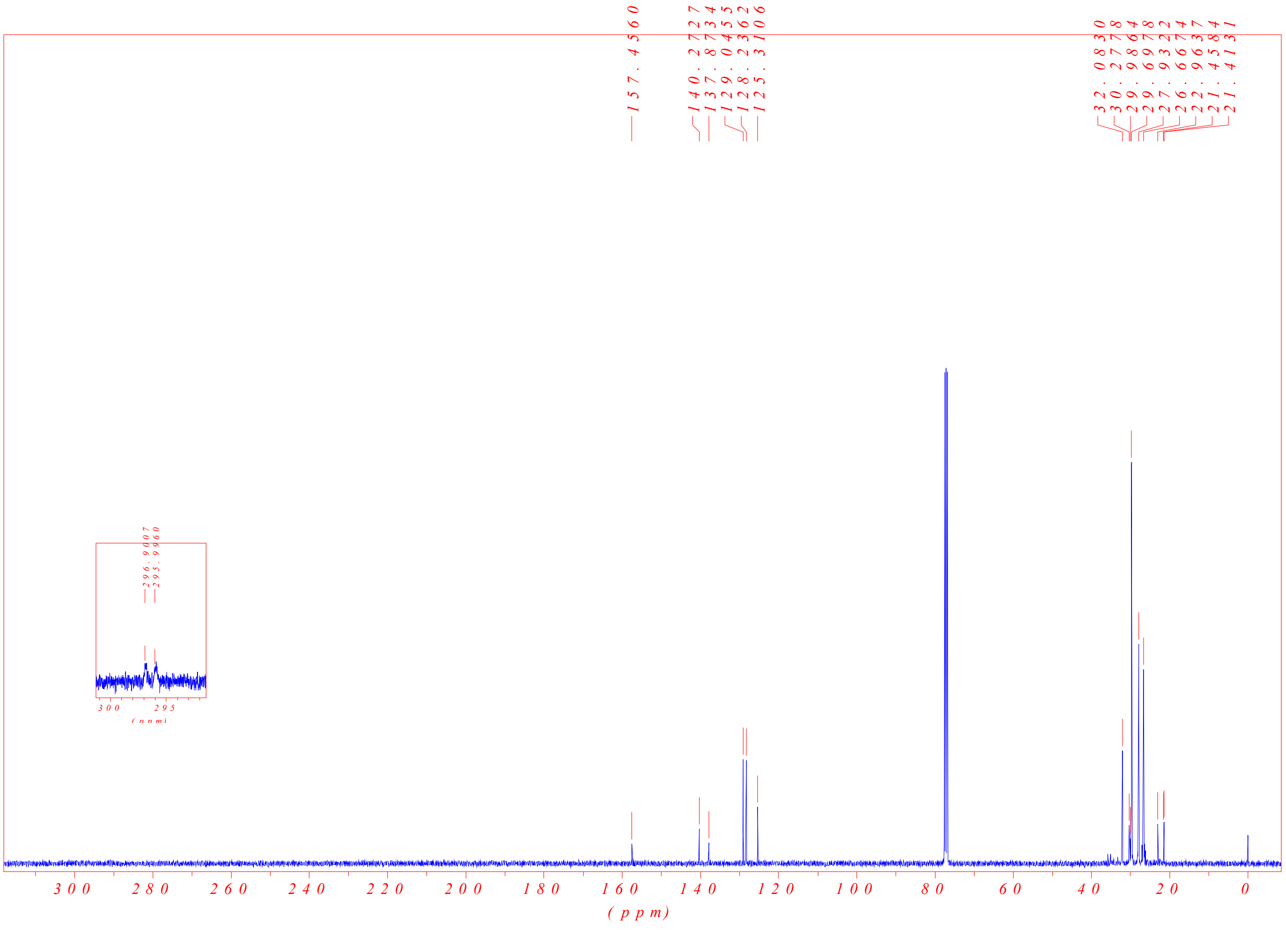

Dichlorobis(tricyclohexylphosphine)(thien-2-ylmethylidene)ruthenium(II) (**1e**):

1H in CD_2Cl_2

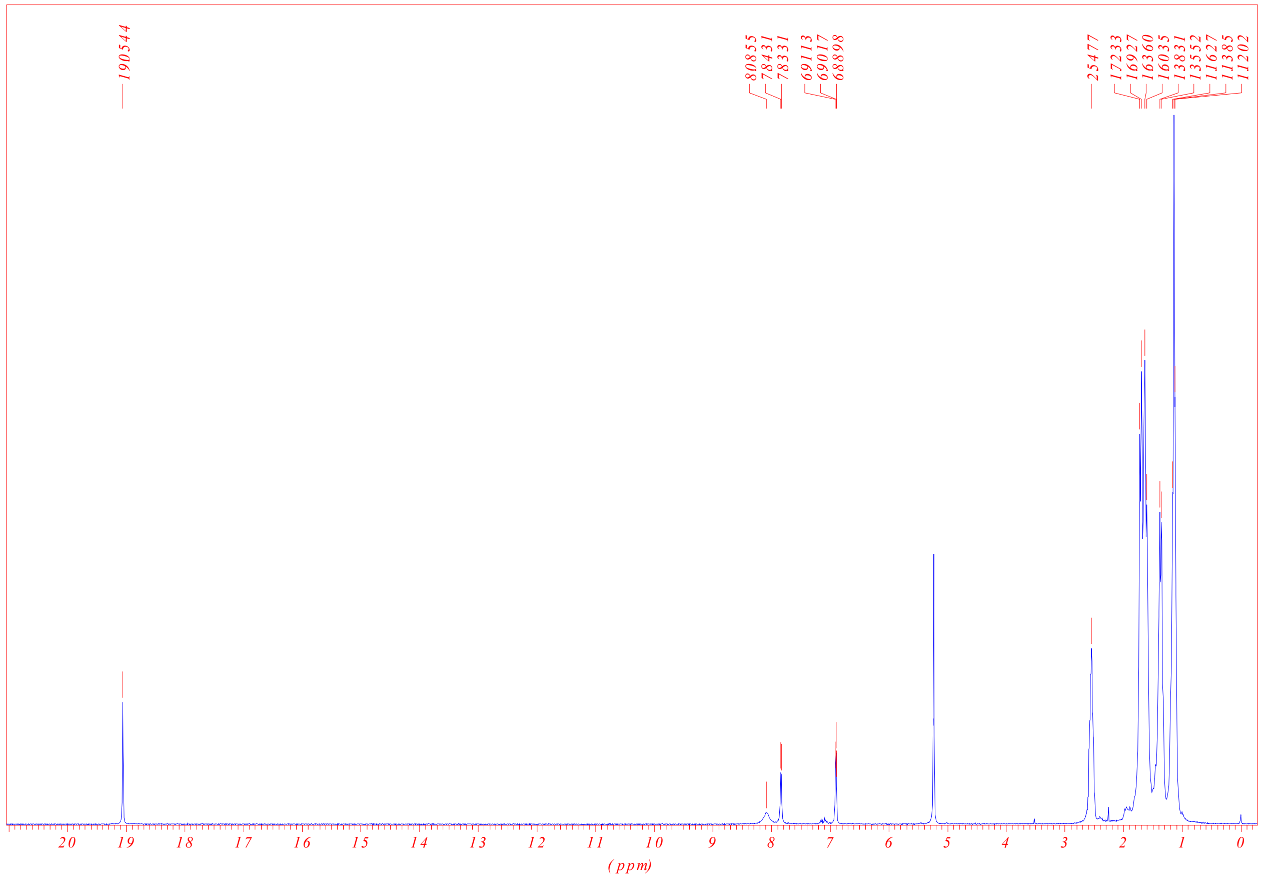

13C in $CDCl_3$

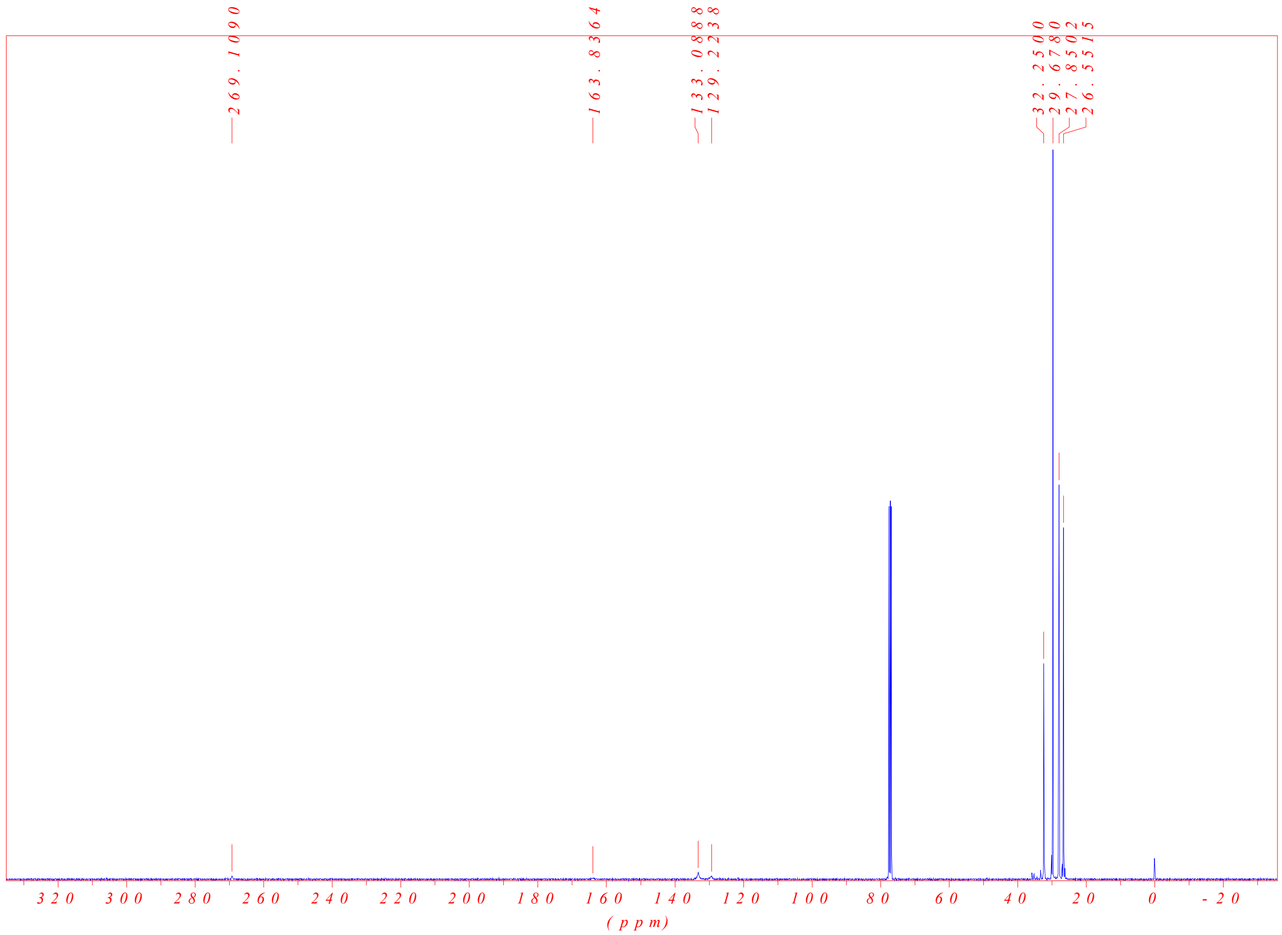

Dichlorobis(tricyclohexylphosphine)(fur-2-ylmethylidene)ruthenium(II) (**1f**):

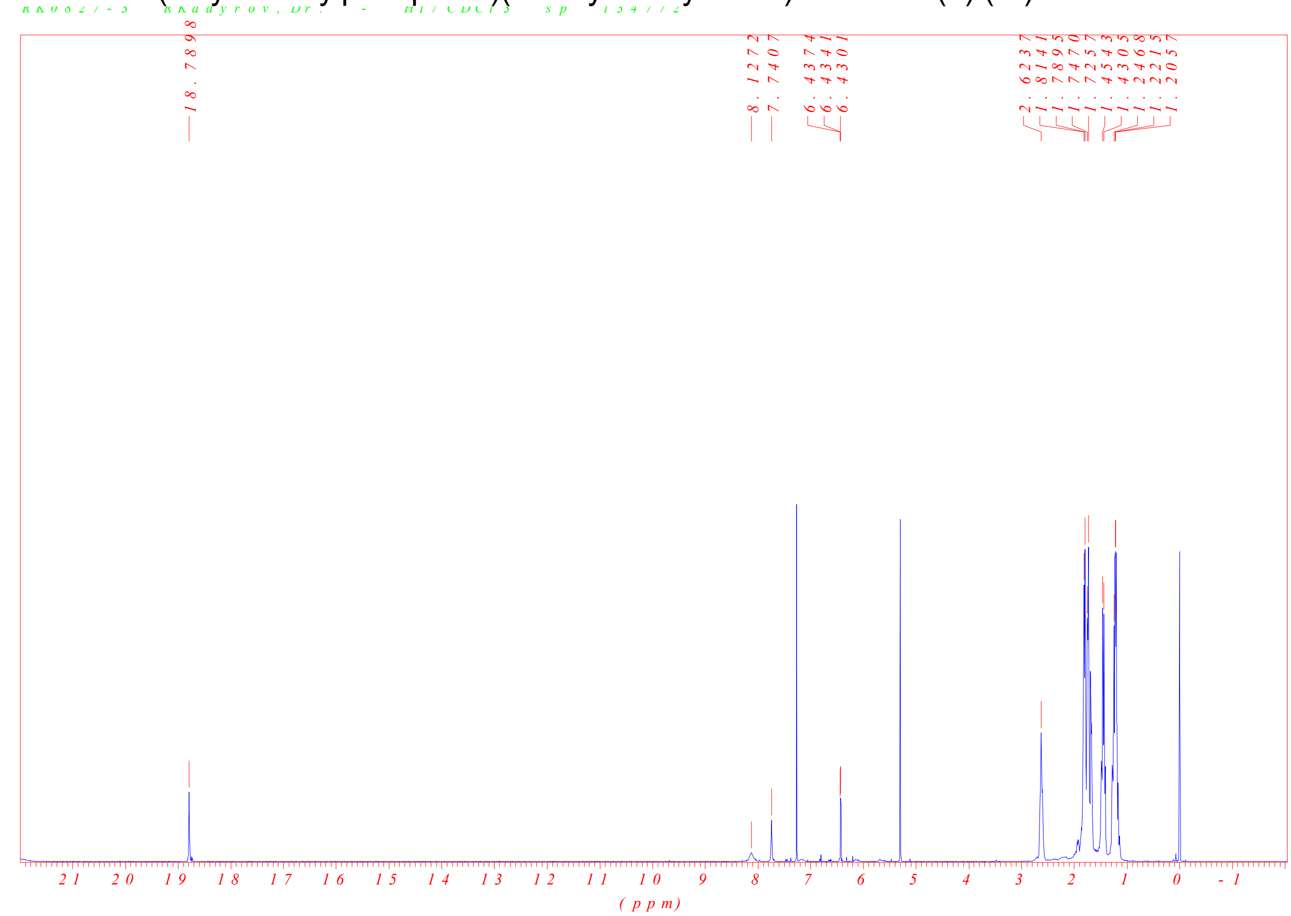

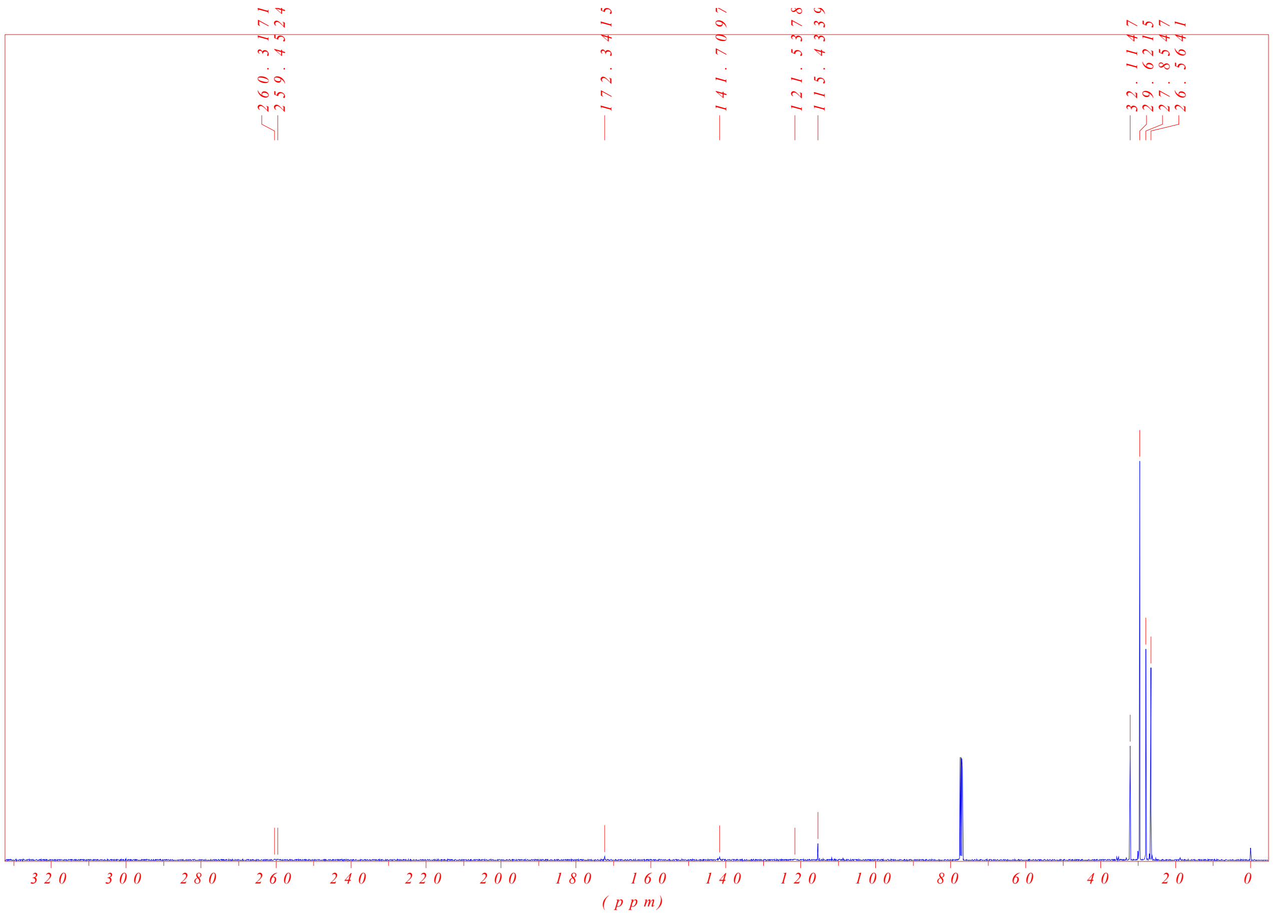

Dichlorobis(tricyclohexylphosphine)(naphth-2-ylmethylidene)ruthenium(II) (**1g**):
1H in CD_2Cl_2

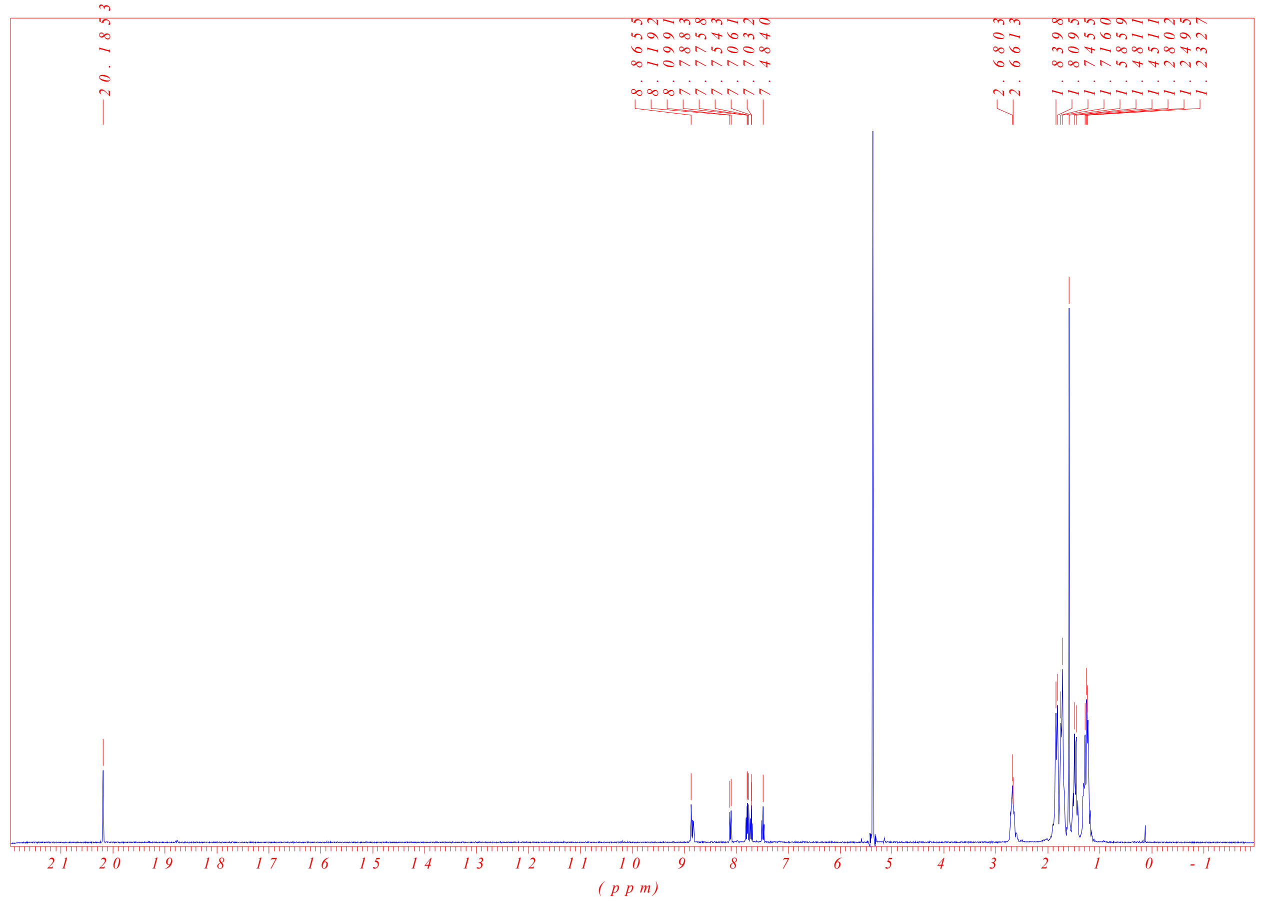

13C in $CDCl_3$

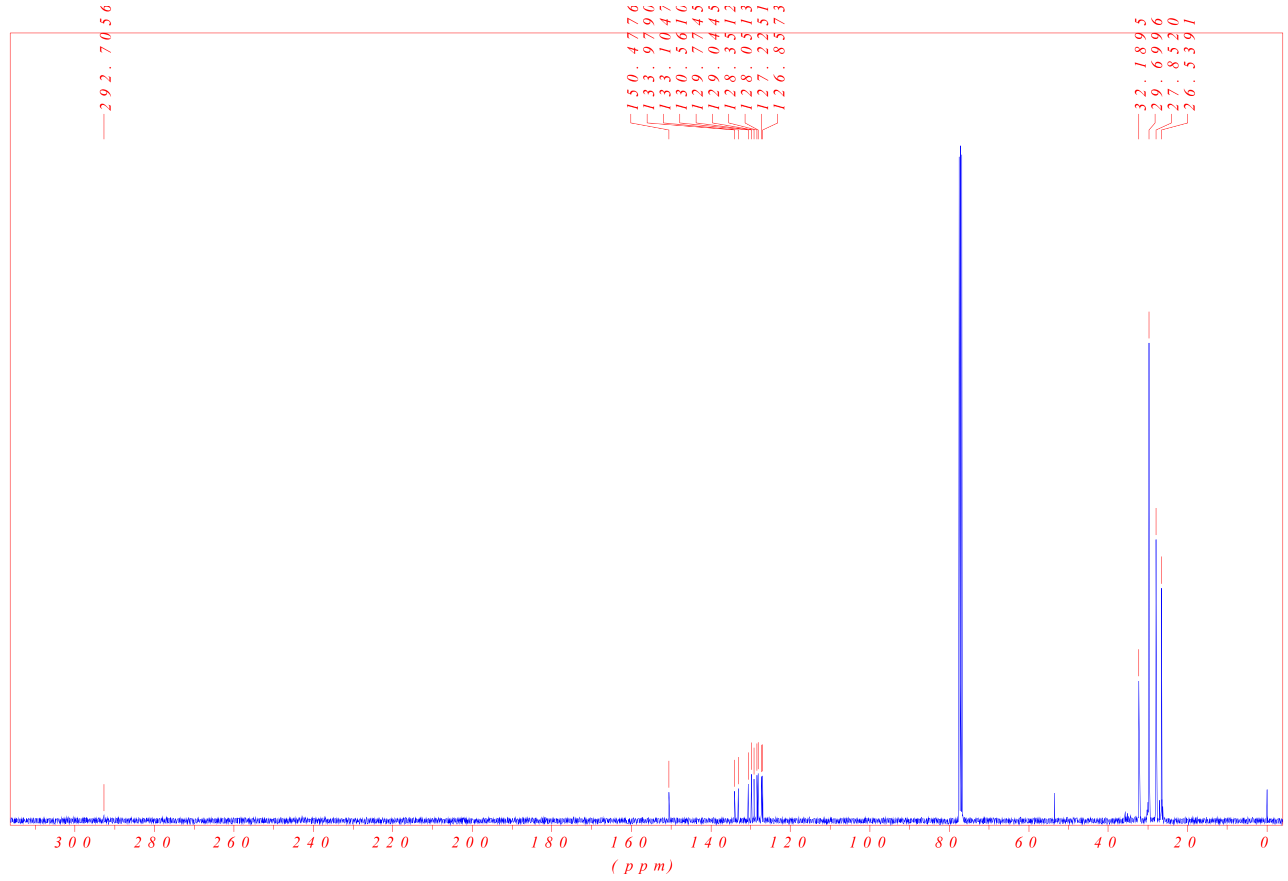

Dichlorobis(tricyclohexylphosphine)(inden-2-ylmethylidene)ruthenium(II) (**1h**):

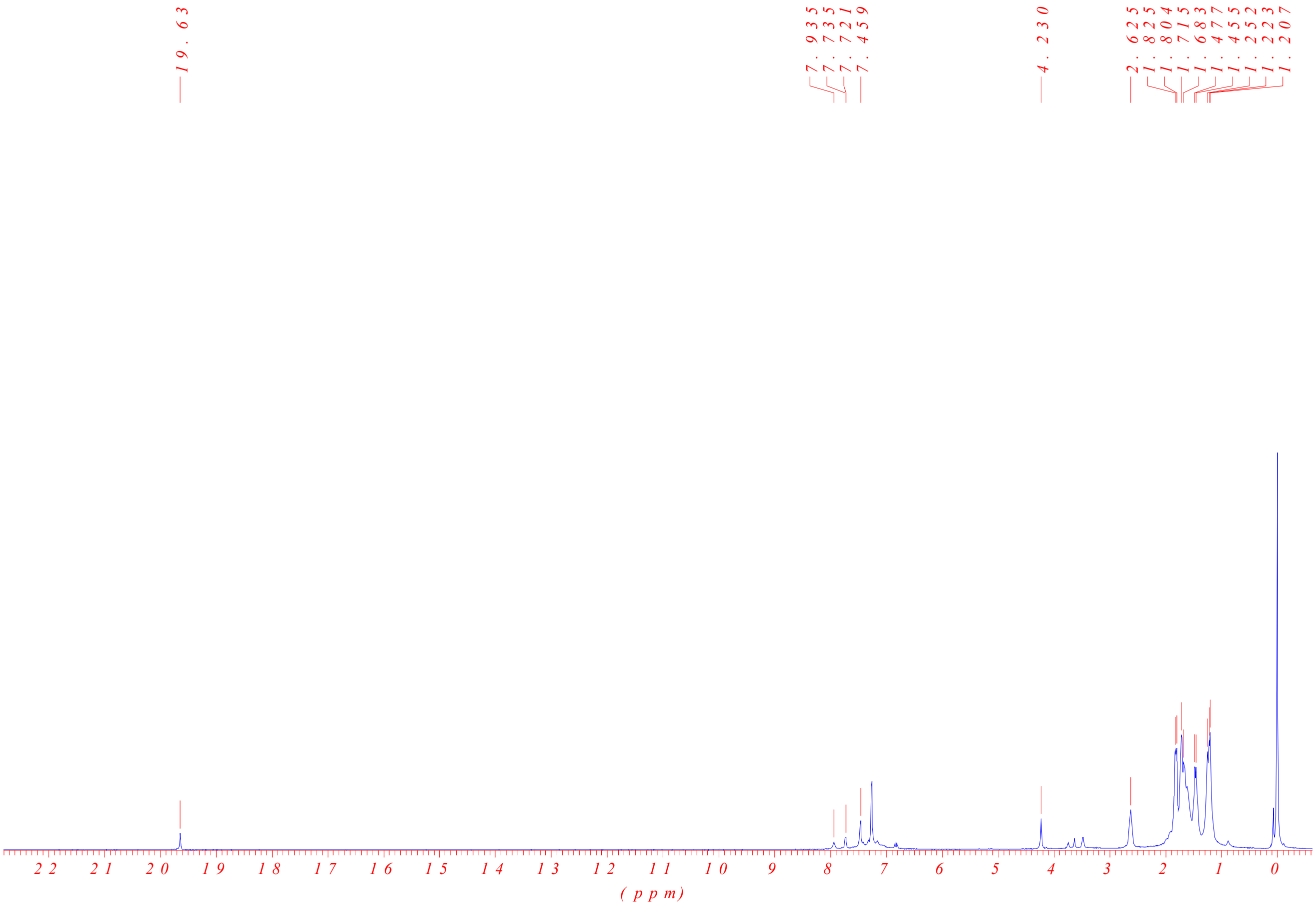

Dichlorobis(tricyclohexylphosphine)(norpinanylmethylidene)ruthenium(II) (**1i**)

1H in CD_2Cl_2

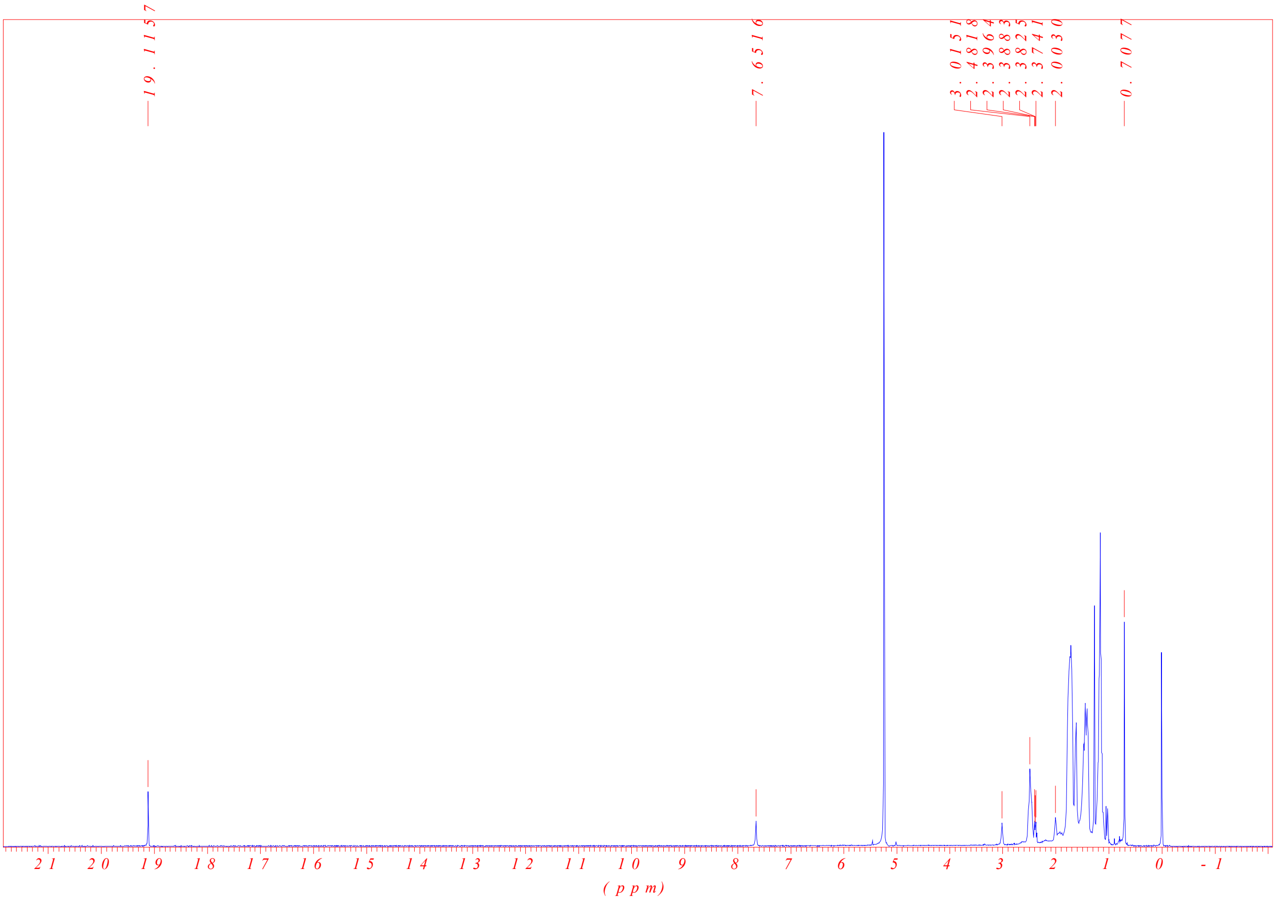

13C in $CDCl_3$

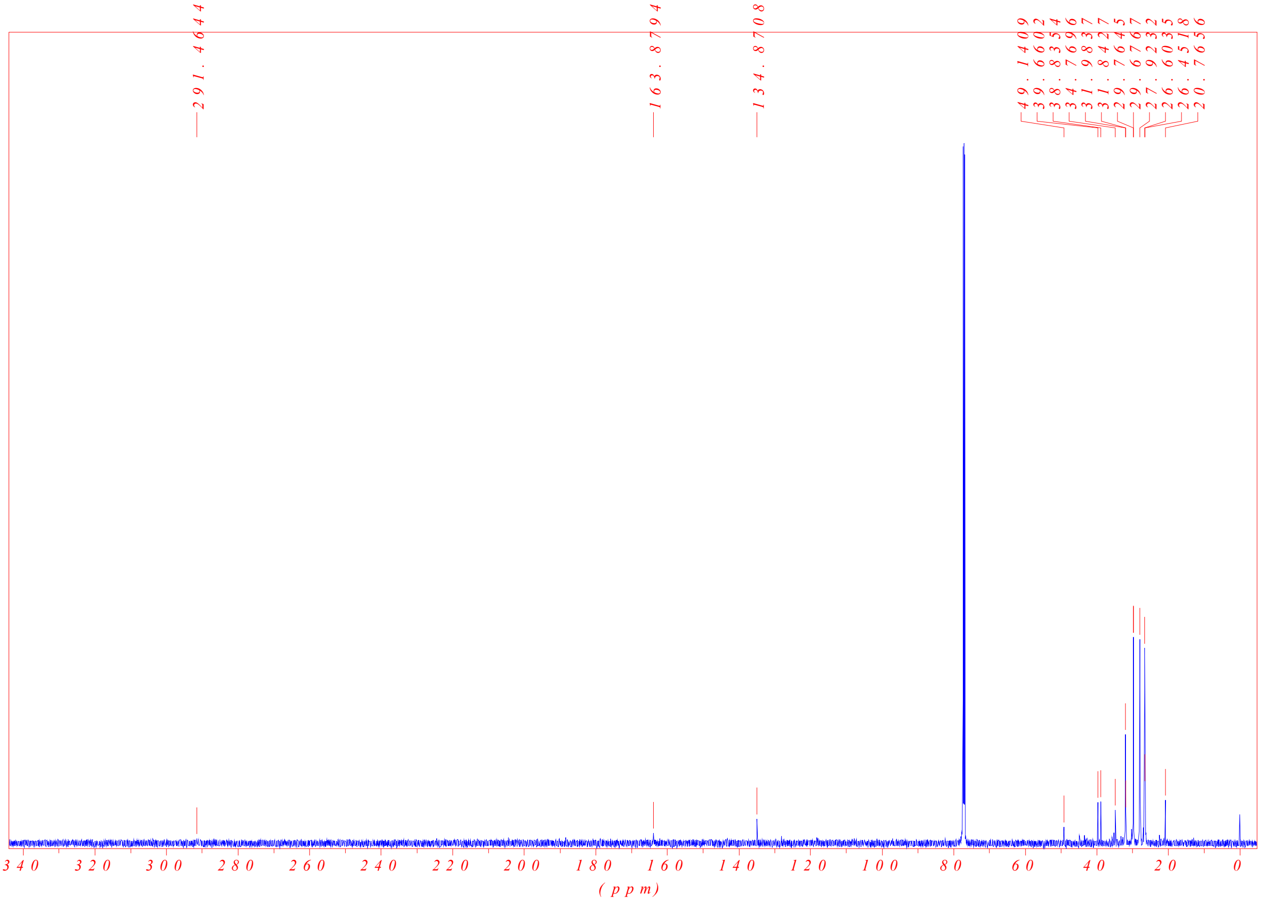

Dichlorobis(tricyclohexylphosphine)(2-phenylvinylylidene)ruthenium(II) (**2**):

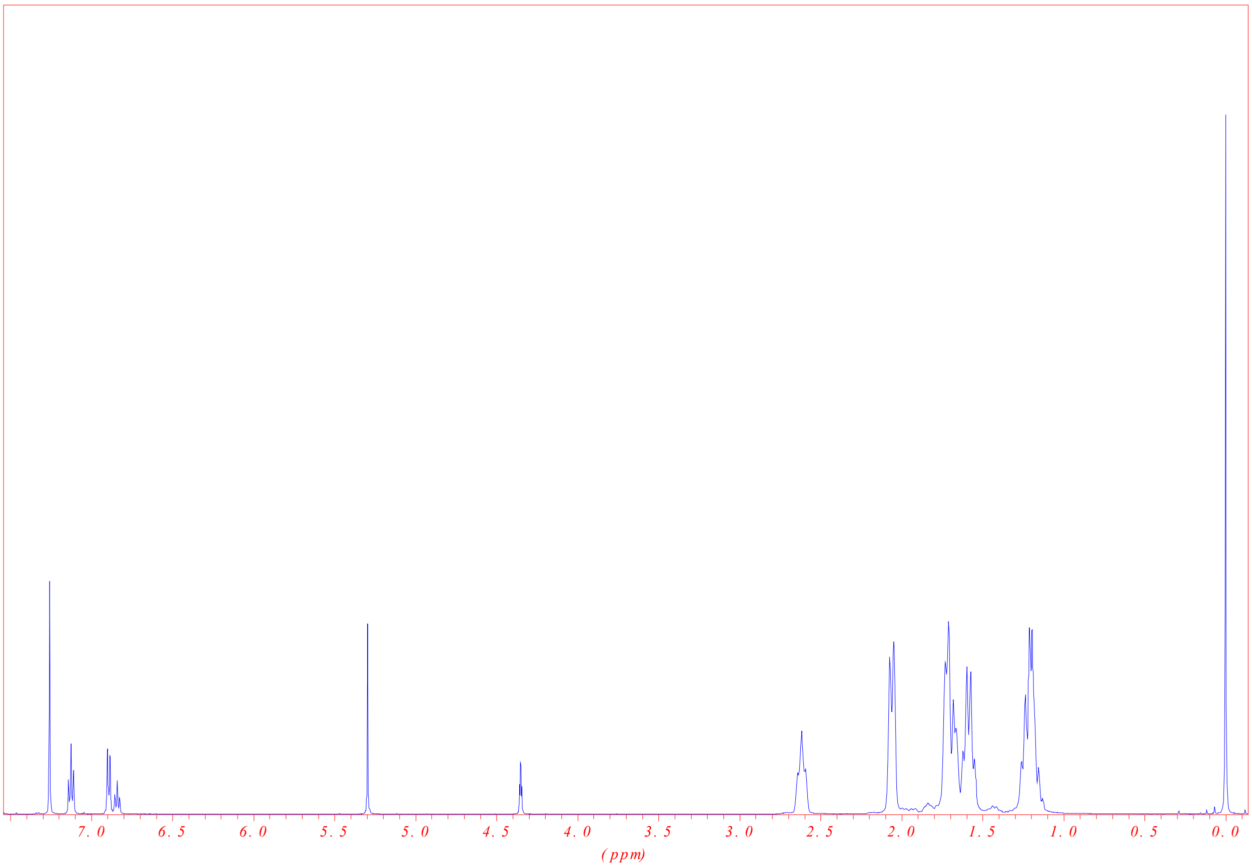

Dichlorobis(tricyclohexylphosphine)(2-butylvinylidene)ruthenium(II) (**3**):

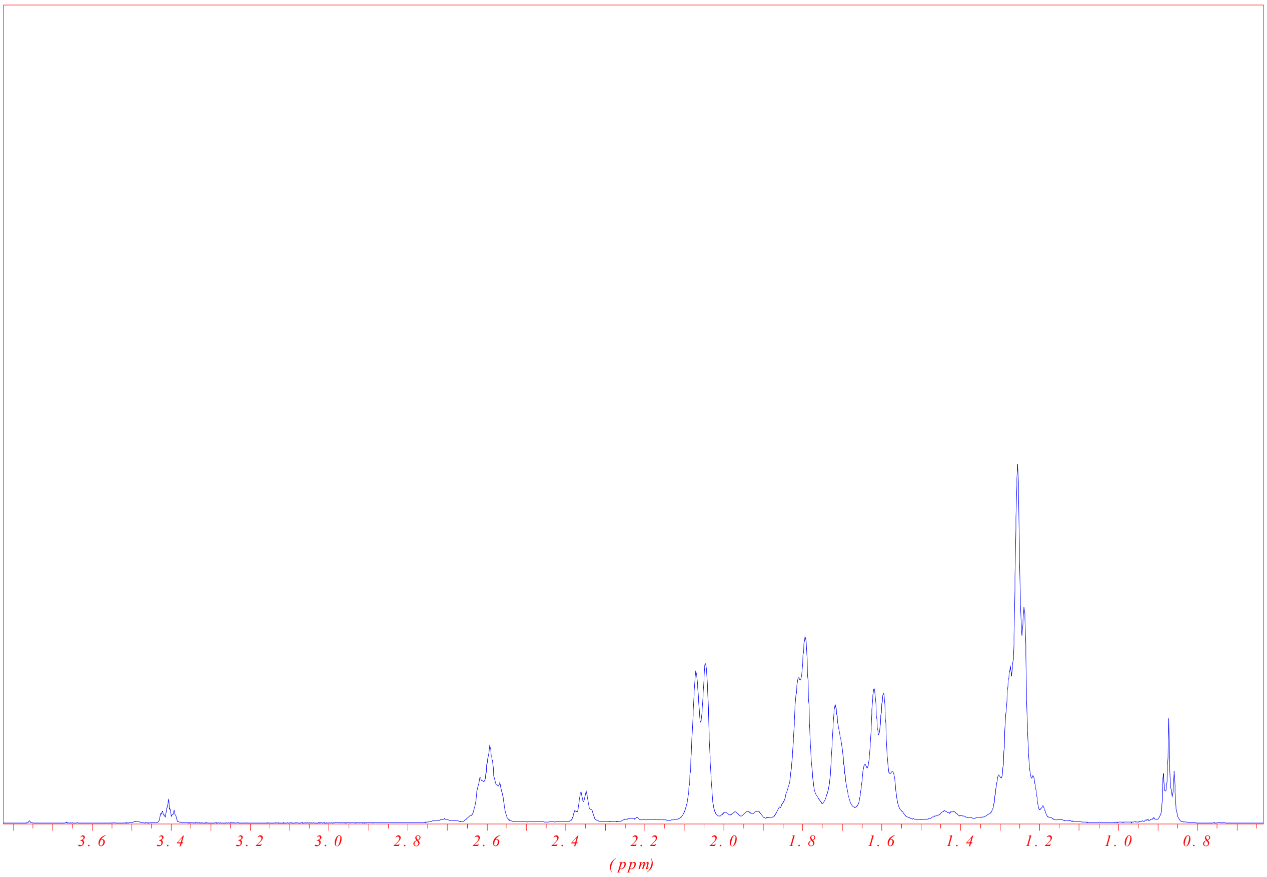

Supplement: File 1 — Detailed experimental data. [file Beilstein_J_Org_Chem-07-104-s001.pdf]
